# A K^+^-sensitive AND-gate dual-mode probe for simultaneous tumor imaging and malignancy identification

**DOI:** 10.1093/nsr/nwac080

**Published:** 2022-04-28

**Authors:** Qiyue Wang, Fangyuan Li, Zeyu Liang, Hongwei Liao, Bo Zhang, Peihua Lin, Xun Liu, Shen Hu, Jiyoung Lee, Daishun Ling

**Affiliations:** Institute of Pharmaceutics, Hangzhou Institute of Innovative Medicine, College of Pharmaceutical Sciences, Zhejiang University, Hangzhou 310058, China; Institute of Pharmaceutics, Hangzhou Institute of Innovative Medicine, College of Pharmaceutical Sciences, Zhejiang University, Hangzhou 310058, China; World Laureates Association (WLA) Laboratories, Shanghai 201203, China; Institute of Pharmaceutics, Hangzhou Institute of Innovative Medicine, College of Pharmaceutical Sciences, Zhejiang University, Hangzhou 310058, China; Institute of Pharmaceutics, Hangzhou Institute of Innovative Medicine, College of Pharmaceutical Sciences, Zhejiang University, Hangzhou 310058, China; Frontiers Science Center for Transformative Molecules, School of Chemistry and Chemical Engineering, National Center for Translational Medicine, Shanghai Jiao Tong University, Shanghai 200240, China; World Laureates Association (WLA) Laboratories, Shanghai 201203, China; Institute of Pharmaceutics, Hangzhou Institute of Innovative Medicine, College of Pharmaceutical Sciences, Zhejiang University, Hangzhou 310058, China; Frontiers Science Center for Transformative Molecules, School of Chemistry and Chemical Engineering, National Center for Translational Medicine, Shanghai Jiao Tong University, Shanghai 200240, China; World Laureates Association (WLA) Laboratories, Shanghai 201203, China; Department of Obstetrics, The Second Affiliated Hospital of Zhejiang University School of Medicine, Hangzhou 310000, China; Institute of Pharmaceutics, Hangzhou Institute of Innovative Medicine, College of Pharmaceutical Sciences, Zhejiang University, Hangzhou 310058, China; Institute of Pharmaceutics, Hangzhou Institute of Innovative Medicine, College of Pharmaceutical Sciences, Zhejiang University, Hangzhou 310058, China; Frontiers Science Center for Transformative Molecules, School of Chemistry and Chemical Engineering, National Center for Translational Medicine, Shanghai Jiao Tong University, Shanghai 200240, China; World Laureates Association (WLA) Laboratories, Shanghai 201203, China

**Keywords:** dual-mode imaging probe, fluorescence imaging, MRI, tumor malignancy identification, potassium ion

## Abstract

Although molecular imaging probes have the potential to non-invasively diagnose a tumor, imaging probes that can detect a tumor and simultaneously identify tumor malignancy remain elusive. Here, we demonstrate a potassium ion (K^+^) sensitive dual-mode nanoprobe (KDMN) for non-invasive tumor imaging and malignancy identification, which operates via a cascaded ‘AND’ logic gate controlled by inputs of magnetic resonance imaging (MRI) and fluorescence imaging (FI) signals. We encapsulate commercial K^+^ indicators into the hollow cavities of magnetic mesoporous silica nanoparticles, which are subsequently coated with a K^+^-selective membrane that exclusively permits the passage of K^+^ while excluding other cations. The KDMN can readily accumulate in tumors and enhance the MRI contrast after systemic administration. Spatial information of the tumor lesion is thus accessible via MRI and forms the first layer of the ‘AND’ gate. Meanwhile, the KDMN selectively captures K^+^ and prevents interference from other cations, triggering a K^+^-activated FI signal as the second layer of the ‘AND’ gate in the case of a malignant tumor with a high extracellular K^+^ level. This dual-mode imaging approach effectively eliminates false positive or negative diagnostic results and allows for non-invasive imaging of tumor malignancy with high sensitivity and accuracy.

## Teaser

A potassium ion (K^+^) sensitive dual-mode nanoprobe with superior magnetic resonance contrast effect and K^+^-specific fluorescence imaging performance is developed for non-invasive tumor imaging and malignancy identification.

## INTRODUCTION

Identification of tumor malignancy is essential for cancer diagnosis, and determines further clinical therapeutic decision-making [1]. Currently, tissue biopsy is the gold standard for most malignant tumor identification, which involves complex and invasive procedures that can cause great discomfort to patients and potentially increase the risk of distant metastases [2]. Blood biomarker-based liquid biopsy has emerged as a simple and minimally invasive alternative to tissue biopsy [3], although the small differences in the expression levels of biomarkers between cancer patients and healthy individuals restrict its detection accuracy [4]. Furthermore, neither tissue biopsy nor liquid biopsy can achieve real-time spatiotemporal detection of biomarkers in living systems.

With the development of molecular imaging probes, non-invasive medical imaging modalities, such as magnetic resonance imaging (MRI), fluorescence imaging (FI), computed tomography (CT) and ultrasound, have been widely investigated for cancer diagnosis [5–8]. However, benign and malignant lesions may display similar MRI signals due to their similar morphological characteristics, which lead to the overlap in MRI contrast enhancement kinetics [9,10]. FI often fails to reveal anatomical details *in vivo* due to the limited tissue penetration of light, making it merely available to assist malignancy identification during surgery [6,11]. CT and ultrasound are typically based on the morphological differences between benign and malignant lesions, leading to intra- and inter-reader variability, as well as high false positive or negative rates [12–15]. In fact, due to the intrinsic limitations of each imaging modality, the development of imaging strategies that can achieve sensitive and accurate identification of tumor malignancy is extremely challenging.

By rationally integrating different imaging agents, nanoprobes can be tailored to have versatile properties for multimodal imaging, which could compensate for the weaknesses of each imaging modality [1,16,17]. FI brings the capability to identify tumor-associated biomarkers with high sensitivity [18–20]. MRI is a powerful technique capable of acquiring structural and anatomical details of tumors with high spatial resolution, and it can reinforce the utility of FI by providing anatomic correlation to the functional information provided by FI [21,22]. Considering the unique advantages of each imaging modality, the complementary combination of MRI and FI modalities is especially promising for tumor diagnosis [23–25]. However, to the best of our knowledge, multimodal-imaging-based high-performance imaging and identification of tumor malignancy are thus far not realized due to the lack of rationally designed probes.

Considering that necrotic cell death and overexpressed potassium ion (K^+^) channels are major hallmarks of malignant tumors [26–29], but not benign ones, the extracellular K^+^ concentration ([K^+^]_ex_) is significantly elevated in the malignant tumor microenvironment (∼40 mM) compared to that of benign tissue (∼5 mM) [30,31]. Herein, we conceive an inspired non-invasive imaging strategy to detect tumors and simultaneously identify tumor malignancy by using a K^+^-sensitive, ‘AND’-logic-gate-based MRI-FI dual-mode nanoprobe, termed KDMN. The AND gate is a programmable Boolean logic device typically used in computer science, which could also be utilized to process two orthogonal inputs to produce a specific single output for complex biological targets [32–35]. The KDMN integrates MRI-based examination of anatomical details with FI-based detection of [K^+^]_ex_ levels at the region of interest, enabling sensitive tumor imaging and malignancy identification in a single workflow. Moreover, the AND logic gate of a KDMN enables the self-confirmation of MRI and FI results to ensure the accuracy of the diagnosis.

## RESULTS

### Synthesis and characterization of KDMNs

**Figure 1.**
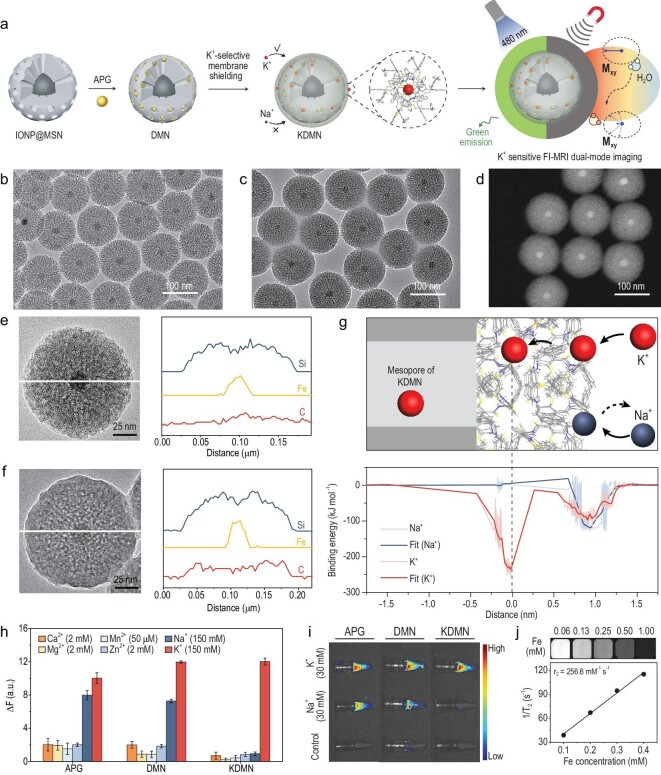
Designed fabrication and characterization of KDMNs. (a) Schematic illustration of the preparation of KDMNs with K^+^-sensitive FI performance and excellent *T*_2_ contrast capability. Transmission electron microscopy (TEM) images of (b) DMNs and (c) KDMNs. (d) Scanning transmission electron microscopy image of KDMNs. High-resolution TEM images and the corresponding EDS elemental line profiles along the white lines of (e) DMN and (f) KDMN. (g) Schematic illustration of the interactions between the filter membrane and K^+^/Na^+^ (upper figure), and the binding energy of K^+^/Na^+^ to the filter membrane (lower figure). (h) Selectivity of the free APGs, DMNs and KDMNs toward K^+^ against other physiological cations. Significant increase in fluorescence intensity of KDMNs is only detected upon addition of 150 mM [K^+^], showing that the KDMNs are highly selective towards K^+^. ΔF = F − F_0_, where F is the fluorescence intensity at a given ion concentration, and F_0_ is the fluorescence intensity without addition of any cations. Data are presented as mean ± s.e.m. (*n* = 3). (i) Fluorescence images of the free APGs, DMNs and KDMNs under different ionic environments. (j) *T*_2_-weighted MRI images and *T*_2_ relaxivity (slope indicates r_2_) of KDMNs.

The KDMNs were prepared by loading commercial K^+^ indicators (Asante Potassium Green-2 tetramethylammonium (TMA^+^) salt, APGs) into the hollow cavities of magnetic mesoporous silica nanoparticles to acquire dual-mode imaging nanoprobes (DMNs), which were further wrapped with a K^+^-selective membrane assembled by three-dimensional (3D) tripodal ligands (1,1,1-tris{[(2^′^-benzyl-aminoformyl)phenoxy]methyl}ethane) (Fig. 1a and Supplementary Fig. 1). The as-prepared KDMNs are highly uniform (Fig. 1b–d), with a hydrodynamic size of 128.5 ± 20 nm and surface charge of −31.8 mV (Supplementary Fig. 2). Energy-dispersive X-ray spectroscopy (EDS) elemental line scanning shows that the carbon element signal derived from the 3D ligands is present in a KDMN but not in a DMN (Fig. 1e and f), indicating the successful assembly of the membrane on the surface of KDMNs, which is also verified by Fourier-transform infrared spectra and thermogravimetric analysis (Supplementary Fig. 3).

We next evaluated the dual-mode imaging performance of KDMNs. The filter membrane of KDMNs can selectively permit the passage of K^+^ while ensuring that other cations, especially sodium ions (Na^+^), do not enter the hollow cavity to activate APGs [36–38], since Na^+^ may bind the aza-crown of APG to induce a false positive fluorescence signal [39]. According to the molecular dynamics simulation results, the binding energy of K^+^ to the pore of the filter membrane is ∼−227 kJ mol^–1^, which is much lower than that of Na^+^ (3.2 kJ mol^–1^), making K^+^ energetically favorable for passing through the filter membrane (Fig. 1g). X-ray photoelectron spectroscopy of KDMNs treated with K^+^ and Na^+^-containing solution reveals obvious K2*P*_3/2_ and K2*P*_1_ binding-energy peaks, but not a Na1*s* binding-energy signal (Supplementary Fig. 4), confirming that only K^+^ can coordinate with 3D ligands and enter the membrane pore. Moreover, among a wider range of different cations, only K^+^ can significantly enhance the fluorescence intensity of KDMNs upon excitation (Fig. 1h). Notably, the KDMNs show the enhanced changes in fluorescence intensity when [K^+^] is increased from 0 to 150 mM, capable of accommodating the variation range of [K^+^]_ex_ in malignant tumors (Supplementary Fig. 5) [30]. In comparison, free APGs or DMNs display poor K^+^ selectivity and can be activated by both K^+^ and Na^+^ (Fig. 1h and i). The K^+^-to-Na^+^ selectivity ratio of the KDMNs is estimated to be ∼14.1, much higher than that of free APGs or DMNs (Fig. 1h). These results demonstrate the excellent selectivity of KDMNs for FI of K^+^, which is essential to excluding interference from other physiological cations, especially Na^+^, that are abundant in the extracellular space. As to MRI contrast capability, KDMNs show a transverse relaxivity (r_2_) value of 256.5 mM^–1^s^–1^, completely adequate for *T*_2_-weighted MRI (Fig. 1j).

### Cellular level [K^+^]_ex_ monitoring and MRI using KDMNs

**Figure 2.**
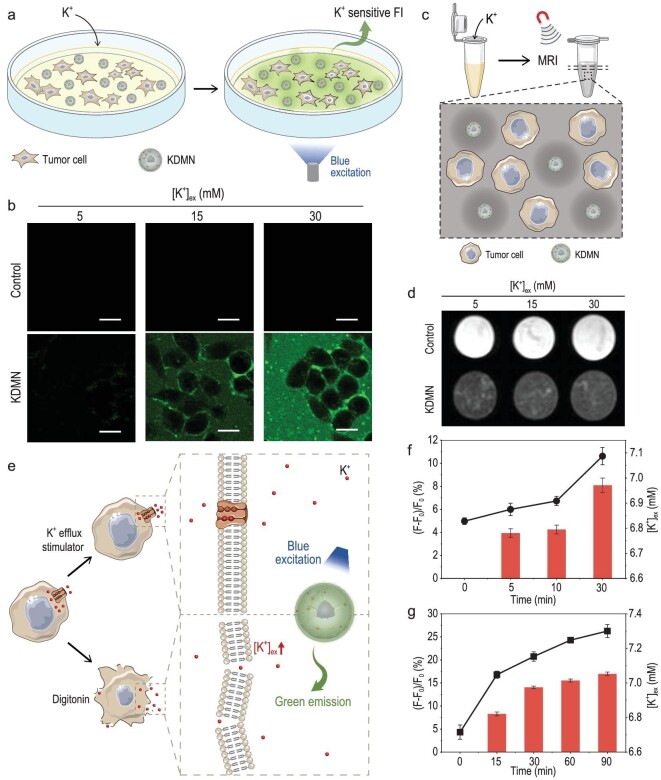
Cellular-level [K^+^]_ex_ monitoring and MRI using KDMNs. (a) Schematic illustration of cellular-level [K^+^]_ex_ monitoring via KDMN-based FI. (b) KDMN-based FI of 4T1 cells in culture medium with different [K^+^] (scale bars = 15 *μ*m). (c) Schematic illustration of cellular level MRI using KDMNs. (d) KDMN-enhanced *T*_2_-weighted MRI of 4T1 cells in culture medium with different [K^+^]. (e) Schematic illustration of the [K^+^]_ex_ monitoring using KDMNs upon addition of K^+^ efflux stimulator or digitonin to induce cell death. The increase in [K^+^]_ex_ of 4T1 cells in response to the (f) K^+^ efflux stimulator and (g) digitonin were determined by measuring the fluorescence intensity changes of KDMNs (histogram) and quantifying [K^+^] using an atomic absorption spectrophotometer (black line). Data are presented as mean ± s.e.m. (*n* = 3).

We further examined the performance of KDMNs in monitoring [K^+^]_ex_ fluctuation of living cells. With the increasing of [K^+^] in the culture medium, the fluorescence signals of KDMNs show a corresponding enhancement outside the cells, indicating that KDMN-assisted [K^+^]_ex_ detection can effectively exclude interference from high intracellular [K^+^] (Fig. 2a and b, Supplementary Figs 6a and 7a). The MRI contrast effect of KDMNs will not be influenced by the interaction with K^+^ (Fig. 2c and d, Supplementary Figs 6b and 7b), guaranteeing congruent MRI performance in different conditions with varying [K^+^]. Moreover, the [K^+^]_ex_ can be increased by treating the cells with the K^+^ efflux stimulator (a mixture of nigericin, bumetanide and ouabain) [40] or with digitonin to increase plasma membrane permeability and induce cell death (Fig. 2e) [41]. Upon drug stimulation, the fluorescence intensities of KDMNs in extracellular space gradually increase over time, reflecting the increase of [K^+^]_ex_ following K^+^ efflux, which is also confirmed by [K^+^] quantification using an atomic absorption spectrophotometer (Fig. 2f and g; Supplementary Figs 6c and d, and 7c and d).

### Logic operation of KDMN-based tumor imaging and malignancy identification

**Figure 3.**
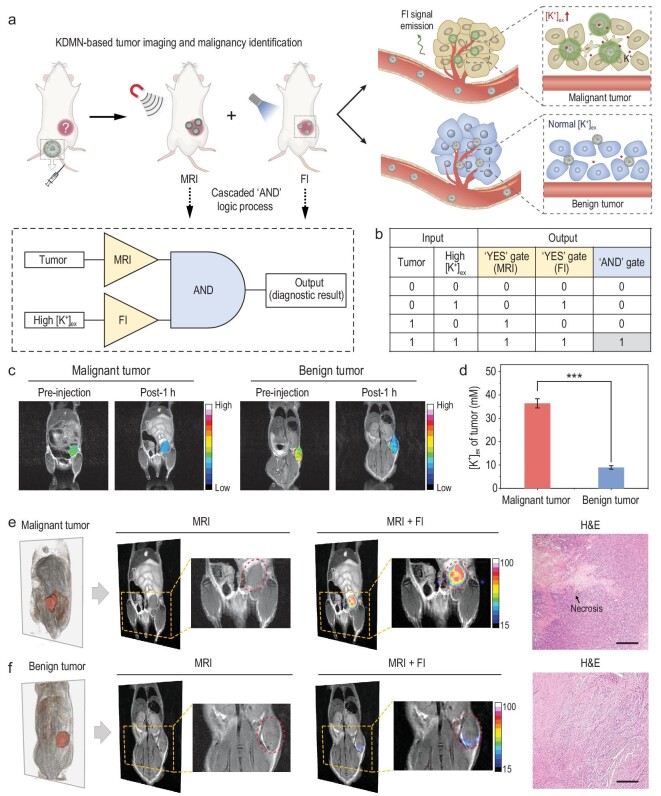
Logic operation of KDMN-based tumor imaging and malignancy identification in living mice. (a) Schematic illustration of KDMN-based AND logic MRI-FI dual-mode imaging for tumor imaging and malignancy identification. (b) The truth table of the cascaded AND logic gate of KDMN. (c) *T*_2_-weighted MRI images of mice bearing malignant or benign xenografts before and after i.v. injection of KDMNs. (d) [K^+^] in TIFs of malignant and benign tumors determined by ICP-MS. Data are presented as mean ± s.e.m. (*n* = 3). Data were compared using unpaired two-tailed Student's t-tests. ^***^P = 0.000202. 3D MRI images, *T*_2_-weighted MRI images, MRI-FI merged images and H&E-stained images (scale bar = 500 *μ*m) of mice bearing (e) malignant and (f) benign xenografts at 1 h after the systemic administration of KDMNs.

The excellent dual-mode imaging performance of KDMNs encouraged us to introduce the highly programmable logic device into the process of tumor imaging and malignancy identification. For the Boolean logic device of a KDMN, the first level of the cascaded logic circuit is composed of two parallel ‘YES’ gates based on KDMN-enhanced MRI and FI, whose outputs are further appointed as the inputs of the second-level AND logic operation (Fig. 3a and b). On the one hand, the KDMN-enhanced MRI-based YES gate outputs 1 in the presence of tumors; on the other hand, in the K^+^ rich extracellular environment of malignant tumors, the KDMNs can trigger a significant fluorescence signal enhancement and give an output 1 in the FI-based YES gate. The final output of the logic gate, which is the diagnostic result, signals a malignant tumor only if the second-level AND gate outputs 1. This cascaded AND logic operation enables a self-confirmation of dual-mode imaging results acquired from KDMN-enhanced structural MRI and functional FI, and thus has the potential to bring the accuracy of tumor malignancy identification to a higher level.

To demonstrate the capability of KDMNs to detect tumors and simultaneously identify tumor malignancy, we further evaluated the imaging performance of KDMNs in mice bearing malignant 4T1 or benign human uterine leiomyoma xenografts. KDMN-based FI is effective in imaging [K^+^]_ex_ change at tumor sites but lacks anatomical information, while KDMN-enhanced MRI offers anatomical images with high soft-tissue resolution that can effectively overcome the intrinsic limitations of FI [25]. After intravenous (i.v*.*) injection, KDMNs firstly confer attenuated *T*_2_-weighted MRI signals at the sites of both malignant and benign tumors, which helps obtain the anatomical location and morphological information of tumors, and give an output 1 in the MRI-based YES gate (Fig. 3c and Supplementary Fig. 8a). Next, we isolated tumor interstitial fluids (TIFs) of the malignant 4T1 xenograft and the benign uterine leiomyoma [30], and compared their [K^+^] levels. The results show that [K^+^] in TIFs from malignant tumors is much higher than that in benign tumors (Fig. 3d), which is consistent with theoretical prediction, demonstrating the feasibility of K^+^ as a biomarker for malignancy identification. Considering that the extent of tumor necrosis has a positive correlation with the aggressive pathological characteristics of the tumor (such as tumor size, stage, grade and so on) [42], we further compared the [K^+^]_ex_ in malignant 4T1 tumor xenografts of different sizes, and found that the level of [K^+^] in TIFs increases with the tumor size (Supplementary Fig. 9). Moreover, *in vivo* FI of [K^+^]_ex_ showed a significant signal enhancement for malignant tumors after KDMN administration, thus generating output 1 in the FI-based YES logic operation (Fig. 3e, and Supplementary Figs 8b and 10). This was in stark contrast to the benign tumors with nearly no changes in fluorescence signal after KDMN administration (FI output = 0) (Fig. 3f, Supplementary Figs 8b and 10). Inductively coupled plasma mass spectrometry (ICP-MS) results further verified the tumor accumulation of KDMNs (Supplementary Fig. 11). Moreover, as shown in hematoxylin and eosin (H&E) staining, necrotic regions that would cause elevated [K^+^]_ex_ are only present in malignant tumors but not in benign ones (Fig. 3e and f), which is in line with their differences in FI signals after KDMN injection. As for the spleen with locally high [K^+^]_ex_, the FI-based logic gate generates the output 1 due to the enhanced fluorescence signal after the administration of KDMNs, while the MRI-based logic gate produces the output 0 because there is no tumor lesion (Supplementary Fig. 12). Only if both dual-mode imaging based YES logic gates output 1, the cascaded AND logic operation will safely output a diagnostic result of malignant tumor. To summarize, KDMN-enabled MRI and FI of [K^+^]_ex_, which is integrated via a cascaded AND logic operation, can simultaneously achieve tumor imaging and malignancy identification with high sensitivity and accuracy.

### Diagnostic accuracy verification of KDMN-based AND logic dual-mode imaging

**Figure 4.**
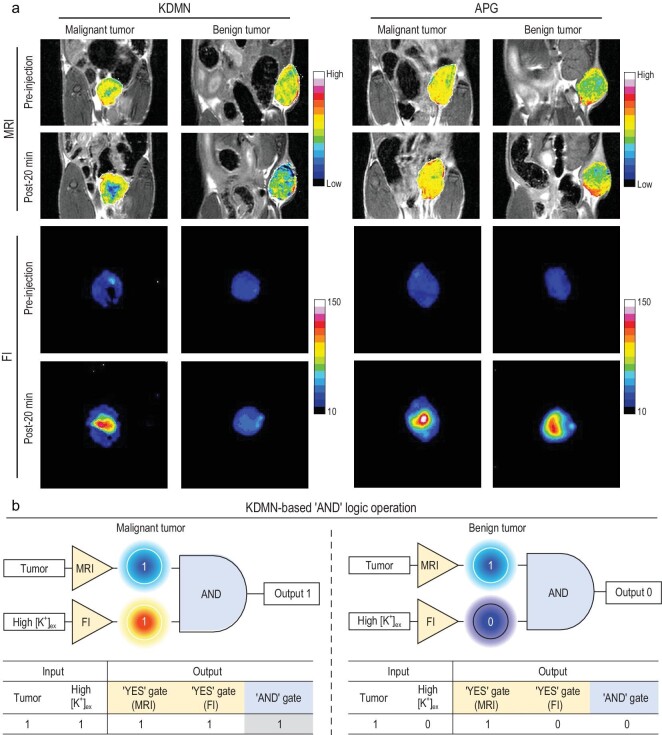
Diagnostic accuracy verification of KDMN-based AND logic dual-mode imaging. (a) MRI and FI images of mice bearing malignant or benign xenografts before and at 20 min after intratumoral injection of KDMNs or free APGs. (b) The symbols and truth tables of the cascaded AND logic gate for processing KDMN-based dual-mode imaging of malignant or benign tumors.

The imaging performance of KDMNs via direct intratumoral injection was investigated to further verify the accuracy of the AND logic imaging strategy. The *T*_2_-weighted MRI signals decrease sharply in both malignant and benign tumors, leading to enhanced imaging contrast and generating outputs 1 in the MRI-based YES gate (Fig. 4a and Supplementary Fig. 13a). Consistent with the imaging results of i.v. administration, only the fluorescence signals for malignant tumors exhibit significant enhancement after intratumoral injection of KDMNs, giving rise to output 1 in the FI-based YES gate (Fig. 4a and Supplementary Fig. 13b). During the second-level AND logic operation, the diagnostic result corresponds to malignant tumors (final output = 1) when the outputs from the MRI- and FI-based YES logic gates are both 1 (Fig. 4b). Compared with KDMNs, free APGs show significantly increased fluorescence signals in both malignant and benign tumors (Fig. 4a), indicating a failure to identify tumor malignancy. This is due to the poor K^+^ selectivity and rapid cellular uptake of free APGs (Fig. 1h and i, Supplementary Fig. 14), which results in severe interference from extracellular Na^+^ and intracellular K^+^. Therefore, we believe that KDMN-based MRI-FI dual-mode imaging and the corresponding cascaded AND logic operation is a plausible strategy for accurate tumor imaging and malignancy identification.

## DISCUSSION AND CONCLUSION

The sensitive imaging and accurate malignancy identification of tumors are significant in clinical cancer management [1]. Presently, plenty of approaches have been developed to evaluate tumor malignancy. For instance, tissue biopsy with high detection sensitivity plays an important role in malignant tumor identification, but its complex and invasive sampling process can cause great discomfort to patients and potentially increase the risk of distant metastases [2]. On the other hand, medical imaging approaches such as MRI, CT and ultrasound have been used for non-invasive tumor diagnosis. Nevertheless, most of these current imaging strategies often depend on imaging probes that lack the level of specificity needed to identify tumor malignancy [9,10,12–15]. Therefore, thus far, to the best of our knowledge, no strategy can realize real-time tumor imaging while simultaneously identifying the malignancy. There remains a need to develop high-performance imaging probes that can improve the sensitivity and accuracy of malignant tumor imaging.

Herein, we reported a K^+^-sensitive AND-gate dual-mode imaging probe, KDMN, that can achieve simultaneous tumor imaging and malignancy identification. With the help of KDMNs, detailed anatomical information obtained by MRI, and K^+^-sensitive fluorescence signals detected by FI, can work in an AND logic gate, and are both required to produce an accurate diagnostic result. Our results show that KDMN-enhanced MRI confers attenuated signals at the tumor sites for effective tumor detection regardless of systemic or local administration. Meanwhile, KDMN-based K^+^-sensitive FI shows a significant difference in fluorescence signals between malignant tumors and benign ones because there is an elevated [K^+^]_ex_ level in the malignant tumor microenvironment [30,31]. Moreover, the integration of KDMN-based MRI and FI via the cascaded logic circuit successfully achieves self-confirmation of dual-mode imaging results, thus allowing more reliable and accurate imaging of tumor malignancy.

Overall, the K^+^-sensitive AND-gate imaging probe presented here provides a paradigm for designing high-performance contrast agents that integrate the complementary strengths of MRI and FI modalities for highly accurate imaging of tumor malignancy. The level of [K^+^]_ex_ in malignant tumors is supposed to be related to the subgroups and development stages of tumors. Generally, the more aggressive the tumor, the higher [K^+^]_ex_ is, as there is a positive correlation between necrosis and the aggressive pathological characteristics of tumors [42]. The K^+^-sensitive AND-gate imaging probe can be used to study the factors that influence the [K^+^]_ex_ of tumors, and has great potential to monitor dynamic tumor progression, therapy and prognosis.

Furthermore, the conceptual advancement of this logic system can be readily extended to other tumor biomarker-activatable FI-MRI bimodal probes to obtain both the structural and functional information of tumors for improved diagnostic efficacy. Despite the success of simultaneous MRI and FI in preclinical studies [43], their clinical application still faces technical challenges, such as the lack of commercially available equipment integrating MRI and FI, which certainly requires more industrial effort. Further development of tissue-penetrating near-infrared dual-mode AND-gate imaging probes, e.g. by integrating upconversion nanoparticles and magnetic nanoparticles, would allow the highly sensitive imaging of K^+^ level in deep-seated tissues with reduced background signal noise [37]. Once immobilized with specific targeting ligands, these imaging probes shall have broad prospects for precise imaging and monitoring of not only malignant tumors, but also many other K^+^-related diseases including neurological disorders and kidney diseases [38,44]. Moreover, metal ion dyshomeostasis is associated with the progression of various major diseases [45,46]. Recently, ion channel detection and regulation have drawn wide attention for medical diagnosis and therapy [47–49]. Based on the established chemical synthetic approach and future development of other specific ion-permeable membranes, it will be practical to further engineer AND-gate dual-mode imaging probes to respond to other metal ions, selectively capturing target ions with well-balanced energetic costs and gains, which might create a new era for developing next-generation imaging probes for highly sensitive and accurate diagnosis of a wide range of ion-dyshomeostasis-associated diseases.

## METHODS

### Synthesis of KDMNs

For the synthesis of iron oxide nanoparticles (IONPs), a mixture of iron-oleate complexes (1.8 g) and oleic acid (0.28 g) was added into 10 g of eicosane, which was degassed at 100°C and then heated up to 343°C under an inert atmosphere. The reaction was maintained at this temperature for 30 min. After cooling the resulting solution to room temperature, acetone was added to precipitate nanoparticles. The collected precipitation was dispersed in 10 mL of chloroform for further use.

The magnetic mesoporous silica nanoparticles were synthesized using a modified Stoöber process; 0.5 mL of IONPs (4 mg mL^–1^) was added dropwise to 5 mL of deionized (DI) water containing 0.1 g of cethyltrimethylammonium bromide (CTAB). The mixture was sonicated for 30 min and then heated to 60°C to evaporate the chloroform. It was then diluted with 45 mL of 0.016 M ammonium hydroxide. Once the mixed solution reached 70°C, 0.5 mL of tetraethyl orthosilicate and 3 mL of ethyl acetate were added immediately and the mixture was kept at that temperature for 3 h under vigorous stirring. The resulting products were collected by centrifugation and washed three times with ethanol. Then, the obtained nanoparticles were dispersed in 50 mL methanol solution (containing 1 wt% sodium chloride) and stirred overnight at 60°C to extract remnant CTAB. After washing with DI water, the magnetic mesoporous silica nanoparticles were redispersed in 5 mL of DI water.

To prepare DMNs, 1 mL of 10 mg mL^–1^ magnetic mesoporous silica nanoparticles and 2 mL of 0.5 mg mL^–1^ free APGs in DI water were mixed and stirred for 24 h at room temperature. The as-synthesized DMNs were collected by centrifugation and washed with DI water, then dispersed in 5 mL of acetonitrile solution for further use.

3D tripodal ligands were prepared as follows: a mixture of N-benzylsalicylamide (3.4 g), anhydrous potassium carbonate (2.5 g) and anhydrous dimethylformamide (25 mL) was heated to 90°C, followed by the addition of 1,1,1-tris(p-tosyloxy-methyl)ethane (2.9 g) and 2-aminoterephthalic acid (0.3 mL). Then, the mixture was stirred for 12 h at 90°C. After cooling to room temperature, the reaction mixture was poured into 200 mL of DI water. The as-synthesized solid products were subjected to a silica gel column using petroleum ether-ethylacetate (2 : 1) as eluent to get 3D ligands as a white solid.

To synthesize KDMNs, 2-mL acetonitrile solution containing 20 mg of 3D ligands was added into a 5-mL DMN acetonitrile ultrasonic suspension (2 mg mL^–1^) under vigorous stirring. The mixed solution was maintained at 50°C for 10 min, followed by annealing at room temperature for 12 h. The resulting nanoprobes were collected by centrifugation and washed with DI water twice, then dispersed in 2 mL of DI water.

## Supplementary Material

nwac080_Supplemental_File
