# Supplementary material for: A K+-sensitive AND-gate dual-mode probe for simultaneous tumor imaging and malignancy identification
Source: Natl Sci Rev. 2022 Apr 28;9(7):nwac080. doi: 10.1093/nsr/nwac080 (PMC9273306; doi:10.1093/nsr/nwac080)
Supplement: nwac080_Supplemental_File [file nwac080_supplemental_file.docx]

**Supplementary Information**

**A K^+^-sensitive AND-gate dual-mode probe for simultaneous tumor imaging and malignancy identification**

Qiyue Wang^1,†^, Fangyuan Li^1,3,†,*^, Zeyu Liang^1^, Hongwei Liao^1^, Bo Zhang^2,3^, Peihua Lin^1^, Xun Liu^2,3^, Shen Hu^4^, Jiyoung Lee^1^ and Daishun Ling^1,2,3,*^

^1^Institute of Pharmaceutics, Hangzhou Institute of Innovative Medicine, College of Pharmaceutical Sciences, Zhejiang University, Hangzhou 310058, China

^2^Frontiers Science Center for Transformative Molecules, School of Chemistry and Chemical Engineering, National Center for Translational Medicine, Shanghai Jiao Tong University, Shanghai 200240, China

^3^WLA Laboratories, Shanghai 201203, China

^4^Department of Obstetrics, The Second Affiliated Hospital of Zhejiang University School of Medicine, Hangzhou 310000, China

^†^These authors contributed equally to this work.

***Corresponding authors.** E-mails: lfy@zju.edu.cn; dsling@sjtu.edu.cn

**Experimental section**

**Materials**. All commercially available chemicals and materials were applied without further purification. Oleic acid, and ammonium hydroxide was purchased from Sigma-Aldrich. Eicosane, cethyltrimethylammonium bromide, tetraethyl orthosilicate, N-benzylsalicylamide, anhydrous dimethylformamide, and 2-aminoterephthalic acid were obtained from Aladdin. Ethylacetate, chloroform, acetone, acetonitrile, methanol, sodium chloride, petroleum ether, and anhydrous potassium carbonate were purchased from Sinopharm Chemical Reagent. Commercial K^+^ indicator (APG) was obtained from Abcam. Deionized (DI) water was prepared by Milli-Q (Millipore, USA). Roswell Park Memorial Institute (RPMI) 1640 medium, trypsin-EDTA solution, and fetal bovine serum were purchased from Jinuo Biomedical Technology. Matrigel was purchased from BD Bioscience.

**Instrumentation.** Transmission electron microscopy images and the corresponding energy-dispersive X-ray spectroscopy elemental line profiles were acquired via a transmission electron microscope (JEOL JEM-2100F, Japan). Dynamic light scattering measurements were performed using a Zetasizer Nano ZS90 instrument (Malvern, U.K.). Fluorescence intensity was recorded on Cary Eclipse (Agilent, U.S.A). Thermogravimetric analysis was acquired via a thermogravimetric analyzer (TA instruments SDT Q600, U.S.A). K^+^ concentration ([K^+^]) in cell culture medium was determined by atomic absorption spectrum instrument (Agilent Technologies 240FS AA, U.S.A). Fluorescence images were collected on a VISQUE InVivo Elite imaging device (Vieworks, Korea). Magnetic resonance imaging (MRI) were performed using a 9-T MRI scanner (Time Medical Systems Nova 9T/110, U.S.A). Fourier-transform infrared (FTIR) spectra were obtained on a FTIR spectrometer (Nicolet iS50, U.S.A). X-ray photoelectron spectroscopy (XPS) analysis was acquired by using an XPS system (Thermo Scientific ESCALAB 250 Xi XPS system, U.S.A). [K^+^] in tumor interstitial fluid (TIF) was quantified by inductively coupled plasma mass spectrometry (ICP-MS, PerkinElmer NexION 300X, U.S.A).

**K^+^-sensitive fluorescence imaging (FI) and MRI performance of nanoprobe.** K^+^-sensitive dual-mode nanoprobes (KDMNs) were dispersed in DI water and the K^+^-sensitive FI performance was evaluated using fluorescence spectrophotometer and InVivo Elite imaging device upon 480 nm excitation or GFP excitation respectively. The MRI performance of KDMNs was investigated using a 9-T MR scanner. Multi-slice multi-echo sequence was used to measure *T*_2_ (repetition time (*T_R_*) = 5000 ms, echo time (*T_E_*) = 12.22, 24.44, 36.66, 48.88, 61.1,73.32 ms, field of view (FOV) = 40 × 40 mm^2^, matrix = 256 × 255, and slice thickness/gap = 1.5 mm/0.1 mm). For *T*_2_-weighted MRI image, the measurement parameters were as follows: *T_R_* = 5000 ms, *T_E_* = 20 ms, FOV = 40 × 40 mm^2^, matrix = 256 × 255, and slice thickness/gap = 1.5 mm/0.1 mm.

**Molecular dynamics simulations.** The structure data of KDMNs membrane was obtained from the crystal structure using X-ray crystal diffraction. The General Amber force field parameters of KDMNs membrane was generated by AmberTools script, and the restrained electrostatic potential charge of the KDMNs membrane was calculated by Gaussian 03 with the Hartree-Fork method using 6-31 + G(d) basis set. The parameters for the solvents and ions were obtained from Amber 03 force field.

GROMACS 5.0 was used to operate system construction and molecular dynamic simulation process, and Visual Molecular Dynamics graphics software was used to visualize the structures and simulation process. The box of the simulation system was set as 6 × 6 × 13 nm^3^ to accommodate KDMNs membrane and proper solvent molecules, with the membrane layer at the center of the box. SP3 water models were added as solvents to fill the box, 20 K^+^/Na^+^ were placed above the membrane layer in water and 20 Cl^-^ were placed below the membrane layer to equilibrate the charge of the system. Then, the construction of the system model was completed.

After the construction of the model, 10-ns molecular dynamics simulation was conducted. The simulation system was set as an isothermal-isobaric ensemble system, with *x* and *y* axis directions were in periodic boundary conditions. The temperature of the system was set as 298 K and the pressure was 1 bar. The time step was set as 2 fs. Electrostatic interactions calculated by the particle mesh Ewald method, and van der Waals interactions was calculated by the Lorentz-Berthelot rule. The cut-off distances of the two interactions above were set as 1.2 nm.

**Extracellular [K^+^] ([K^+^]_ex_) monitoring using KDMNs at the cellular level.** 4T1, B16 and Huh-7 cells were incubated in culture medium with 10% fetal bovine serum at 37℃ under 5% CO_2_. After cells treated with culture medium containing nigericin (5 *μ*M), bumetanide (10 *μ*M), and ouabain (10 *μ*M) or culture medium containing digitonin (5 *μ*M) for varied time points, the [K^+^]_ex_ change was determined by detecting the fluorescence intensity of KDMNs using Cary Eclipse upon 480-nm excitation. Then, the [K^+^]_ex_ was further quantified by atomic absorption spectrum instrument.

**Confocal laser scanning microscopy (CLSM) imaging.** 4T1, B16 and Huh-7 cells (9 × 10^4^ cells per dishes) were cultured on confocal dishes for 24 h. After that, the medium was replaced with 1.5 mL of culture medium containing KDMNs (0.5 mg mL^-1^) under different concentrations of K^+^ (5, 15, 30 mM). After 1 h incubation, the cells were live-imaged by CLSM (Olympus FV1200, Japan).

**Cellular level MRI.** 4T1, B16 and Huh-7 cells were cultured in fresh culture medium or culture medium containing KDMNs (0.5 mg mL^-1^) under different concentrations of K^+^ (5, 15, 30 mM). After incubation for 1 h, the culture medium was collected and the cells were trypsinized. After centrifugation, the cells were re-dispersed in the collected culture medium and stabilized by 1% agarose. For *T*_2_ measurement, *T_R_* = 5000 ms, *T_E_* = 20 ms, FOV = 40 × 40 mm^2^, matrix = 256 × 255, and slice thickness/gap = 1.5 mm/0.1 mm.

**Animal experiments.** BALB/c nude mice (female, 5-6 weeks) were purchased from Shanghai SLAC Laboratory Animal Company, and all of the animal experiments were performed following the procedures of the Institutional Animal Care and Use Committee of Zhejiang University.

**Quantification of [K^+^] in TIFs.** The TIFs of 4T1 tumors with different sizes and uterine leiomyomas were isolated via a centrifugation method. The tumors were harvested and centrifuged at 50 *g* for 5 min to remove surface liquid. Then, samples were centrifuged at 400 *g* for 10 min to obtain TIFs.

**Biodistribution analysis.** The biodistributions of KDMNs were investigated by quantitatively evaluating the amounts of Fe in tumors and main organs before and 1 h after systemic injection of KDMNs (100 mg KDMN kg^-1^, 5 mg Fe kg^-1^). The mice were sacrificed 1 h post-injection, and the tumor tissues as well as main organs, including the heart, liver, spleen, lung and kidney, were removed and weighed. Then, 1 mL of 60% nitric acid was added to digest the tissue samples. The concentrations of Fe were determined by ICP-MS.

***In vivo* dual-mode imaging after intravenous (*i.v.*) injection of KDMNs.** For establishing orthotopic breast cancer mice models, 4T1 cells (10^6^ cells per mouse) were injected into the mammary fat pad of mice. The mice xenograft models of human uterine leiomyoma were established according to the following method. All protocols for human tissue were approved by the Research Ethical Committee of Second Affiliated Hospital, School of Medicine, Zhejiang University. The small pieces of human uterine leiomyoma were implanted with 50 *μ*L of Matrigel in the female mice. The FI of the mice bearing malignant 4T1 or benign uterine leiomyoma xenografts was obtained in the GFP channel of the FI device before and 1 h after *i.v.* injection of KDMNs at a dosage of 100 mg KDMN kg^-1^. After *in vivo* FI of tumors, MRI images were acquired using a fast spin echo sequence: *T_R_* = 1800 ms, *T_E_* = 20 ms, FOV = 50 × 50 mm^2^, matrix of 256 × 255, slice thickness/gap = 0.8 mm/0.1 mm.

***In vivo* dual-mode imaging after intratumor injection of KDMNs or free APGs.** MRI-FI dual-mode imaging was performed before and 20 min after intratumor injection of KDMNs or free APGs, with the same procedures and parameters used in mice models of *i.v.* administration.

**
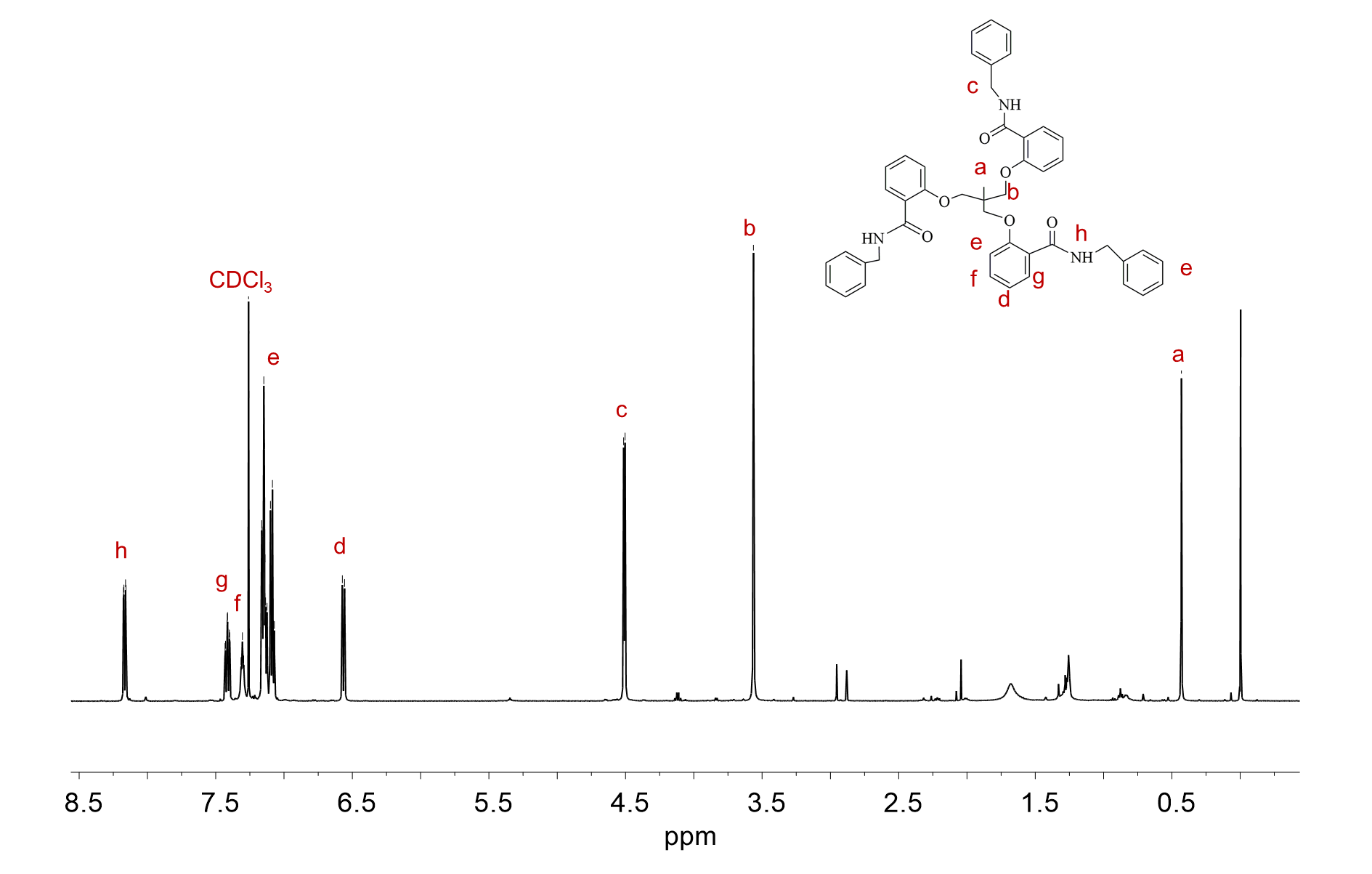
**

**Supplementary Fig. 1.** ^1^H NMR analysis of K^+^-selective 3D ligands. ^1^H NMR spectrum of 3D tripodal ligands in CDCl_3_. *δ*: 0.44 (*s*, 3H, CH_3_), 3.57 (*s*, 6H, OCH_2_), 4.51 (*d*, *J* = 5.2 Hz, 6H, NHCH_2_), 6.57 (*d*, *J* = 8 Hz, 3H, Ar), 7.07-7.16 (*m*, 18H, Ar), 7.26-7.32 (*t*, 3H, Ar), 7.39-7.43 (*dd*, 3H, Ar), 8.15-8.18 (*d*, *J* = 8 Hz, 3H, -NH-).

**
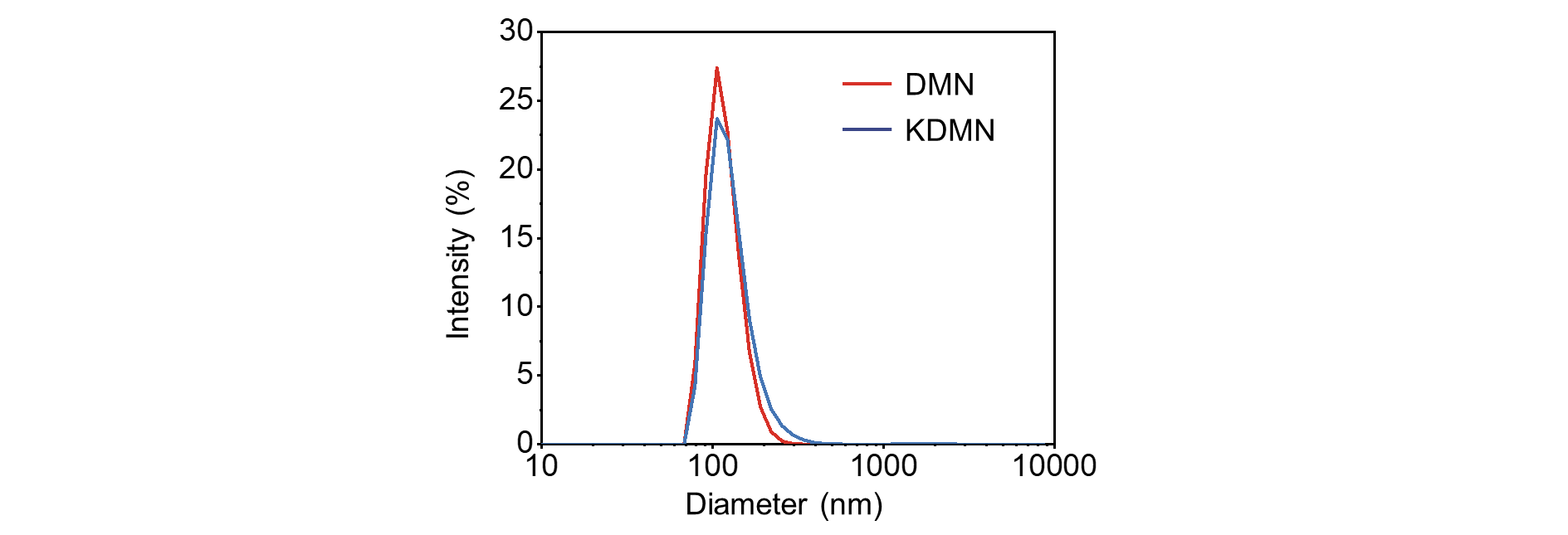
**

**Supplementary Fig. 2.** Size distributions of dual-mode imaging nanoprobes (DMNs) and KDMNs. The hydrodynamic diameter distributions of DMNs and KDMNs.

**
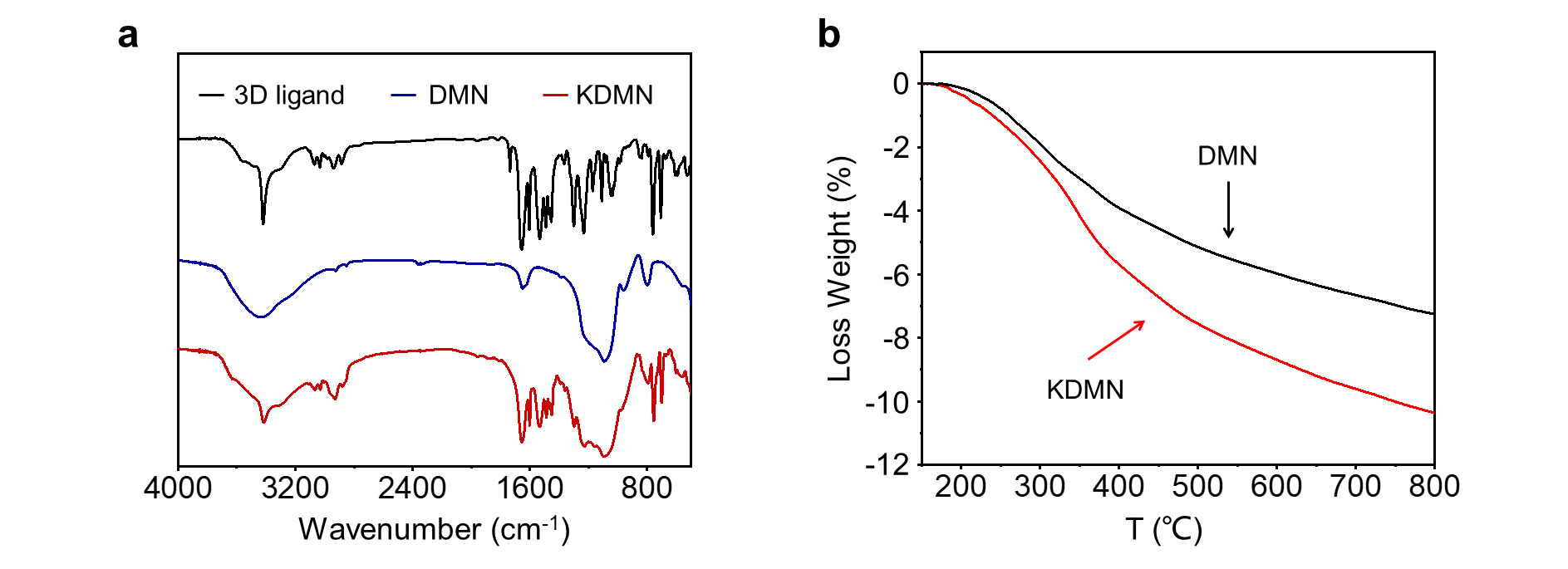
**

**Supplementary Fig. 3.** Characterizations of KDMNs. (a) FTIR spectra of 3D ligands, DMNs and KDMNs. Characteristic bands for 3D ligand are detected in KDMNs, suggesting the successful modification of ligands to KDMNs. (b) Thermogravimetric analysis plots of DMNs and KDMNs. The thermogravimetric results exhibit greater weight loss of KDMNs than DMNs owing to the loss of loaded ligands, which confirm the presence of 3D ligands in KDMNs.


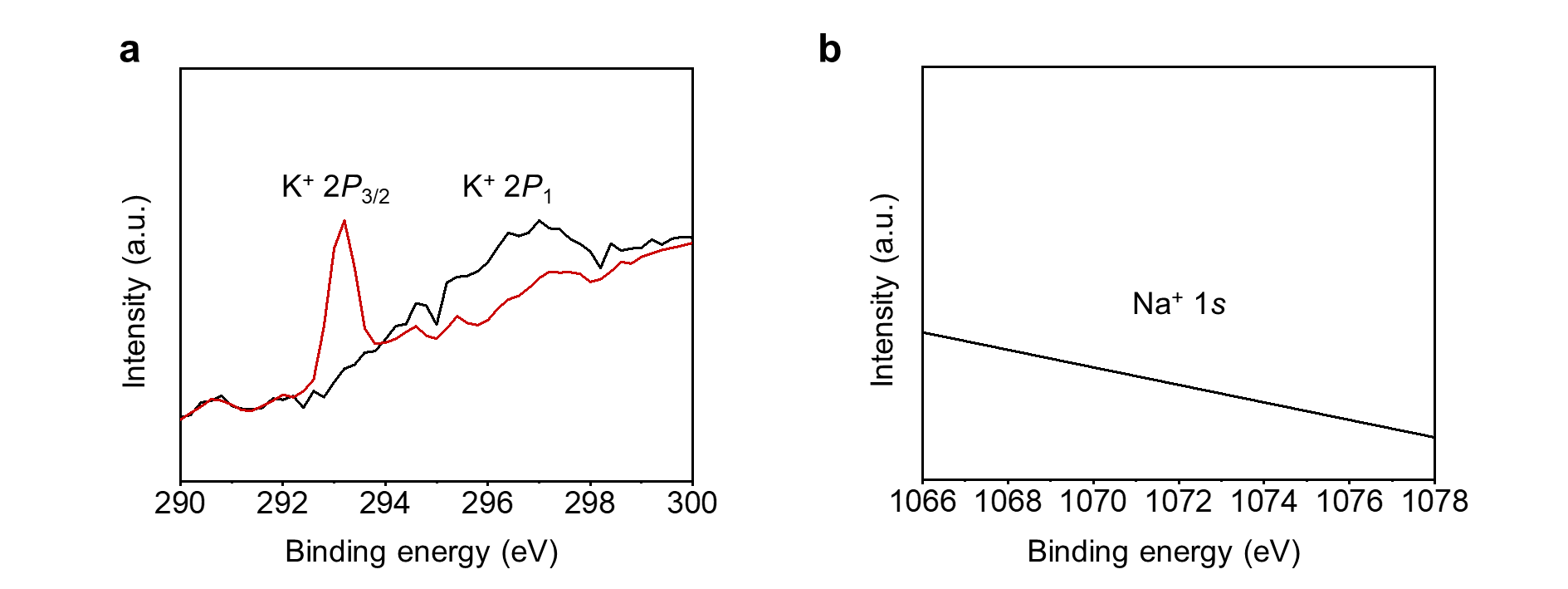


**Supplementary Fig. 4.** XPS analysis of KDMNs after incubation with an aqueous solution containing both Na^+^ and K^+^. (a) The K^+^ 2*P* and (b) Na^+^ 1*s* energy region of the KDMNs after treatment with an aqueous solution containing both Na^+^ and K^+^. The treated KDMNs were washed with the mixed solution of CH_3_OH/H_2_O (v:v = 1:1) before the measurement.


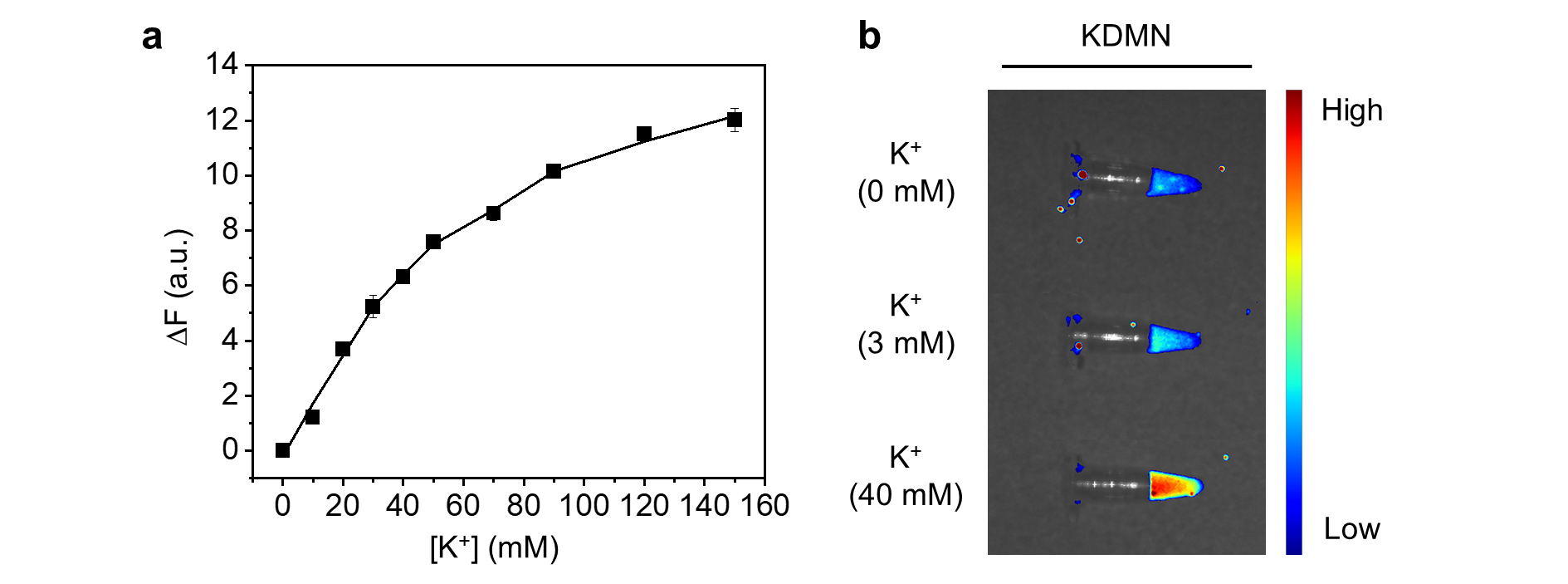


**Supplementary Fig. 5.** K^+^-sensitive FI performance of KDMNs. (a) Changes in the fluorescence intensity of the KDMNs in response to [K^+^] increase from 0 to 150 mM. ΔF = F − F_0_, where F is the fluorescence intensity at a given [K^+^], and F_0_ is the fluorescence intensity without addition of any cations. Data are presented as mean ± s.e.m. (n = 3). (b) *In vitro* FI of KDMNs at different [K^+^].


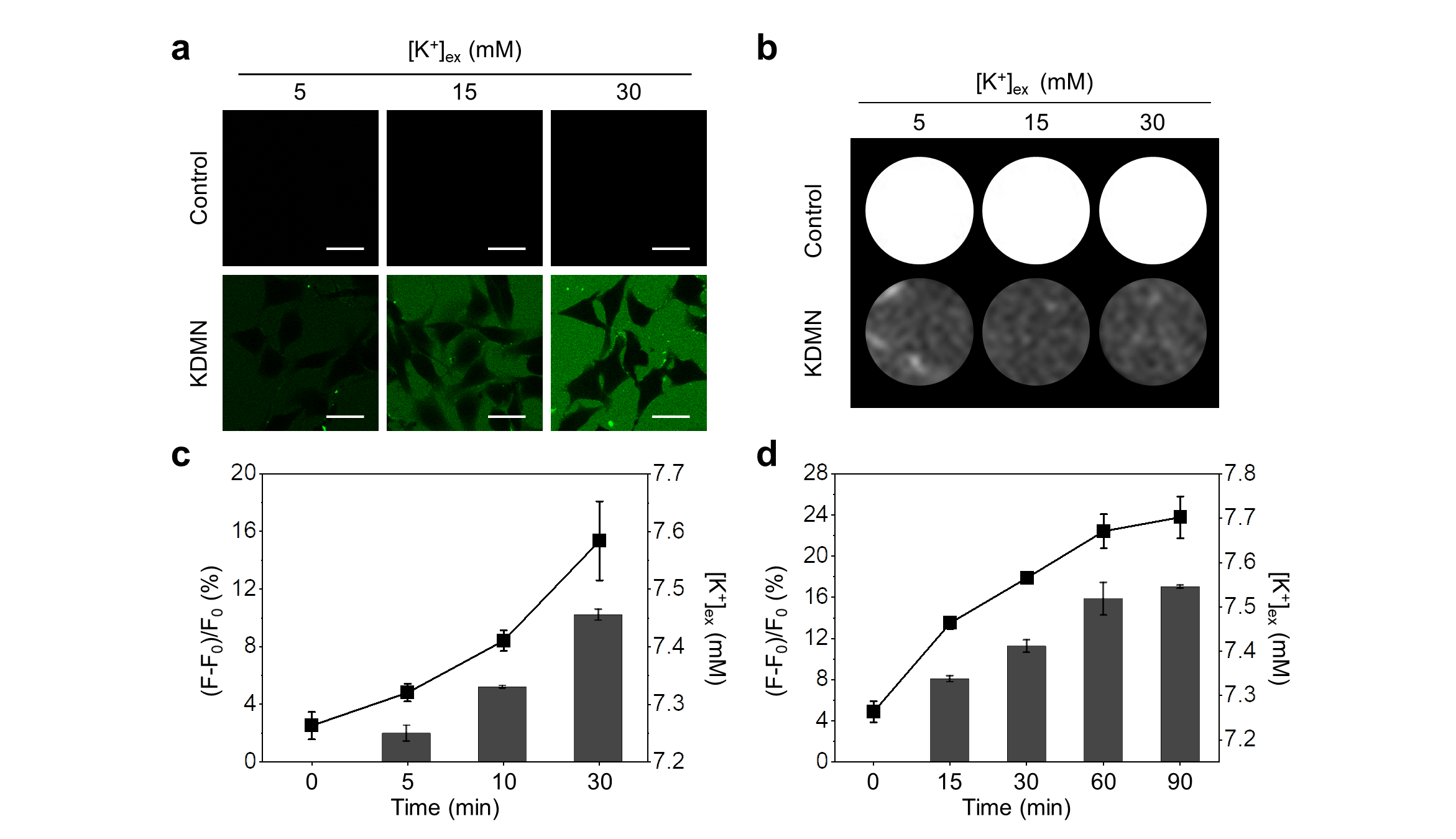


**Supplementary Fig. 6.** (a) KDMN-based FI of B16 cells in culture medium with different [K^+^] (scale bar = 30 *μ*m). (b) KDMN-enhanced *T*_2_-weighted MRI of B16 cells in culture medium with different [K^+^]. The increase in [K^+^]_ex_ of B16 cells in response to the (c) K^+^ efflux stimulator, and (d) digitonin were determined by measuring fluorescence intensity changes of KDMNs (histogram) and quantifying [K^+^] using atomic absorption spectrophotometer (black line). Data are presented as mean ± s.e.m. (n = 3).


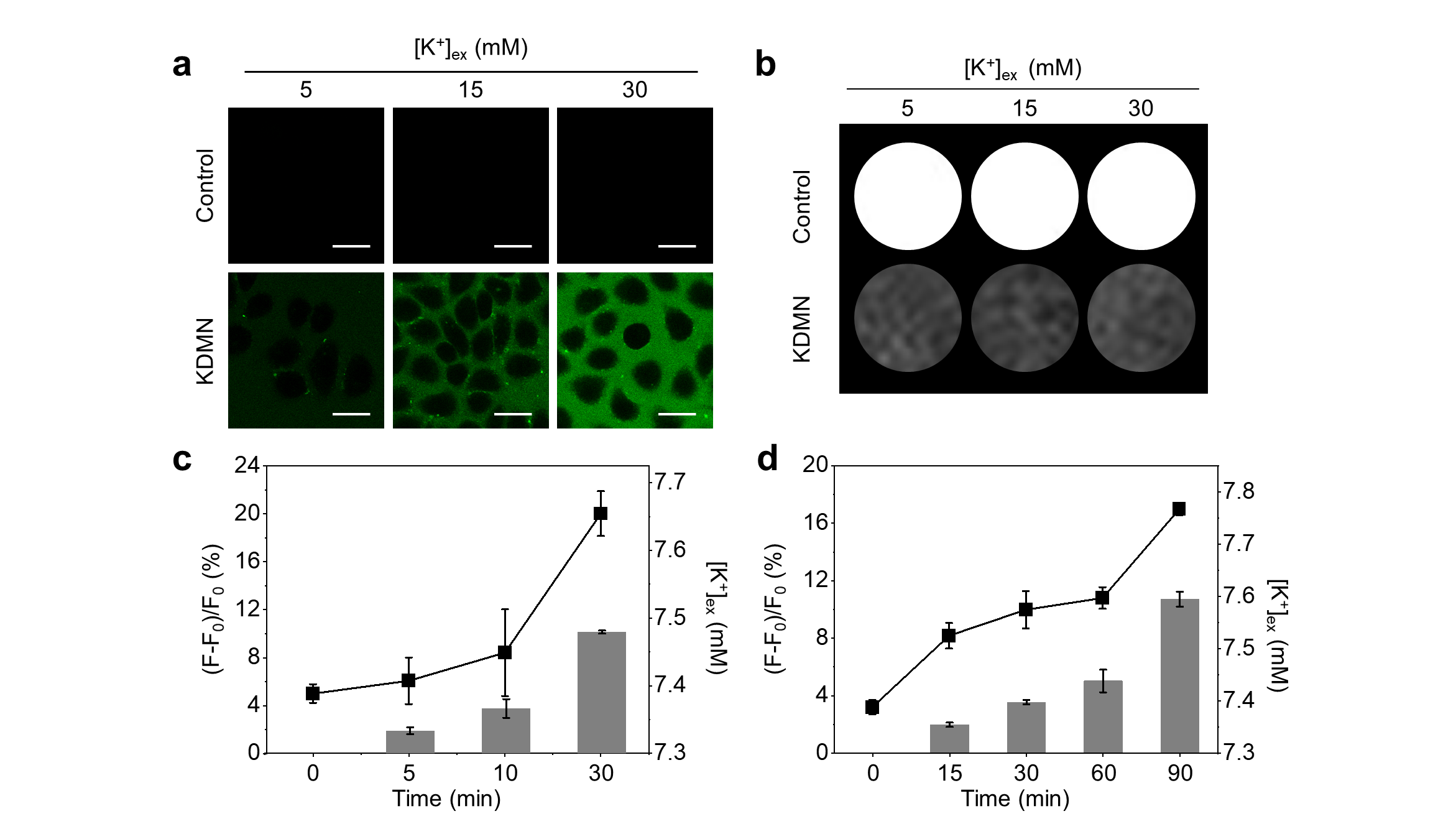


**Supplementary Fig. 7.** (a) KDMN-based FI of Huh-7 cells in culture medium with different [K^+^] (scale bar = 30 *μ*m). (b) KDMN-enhanced *T*_2_-weighted MRI of Huh-7 cells in culture medium with different [K^+^]. The increase in [K^+^]_ex_ of Huh-7 cells in response to the (c) K^+^ efflux stimulator, and (d) digitonin were determined by measuring fluorescence intensity changes of KDMNs (histogram) and quantifying [K^+^] using atomic absorption spectrophotometer (black line). Data are presented as mean ± s.e.m. (n = 3).


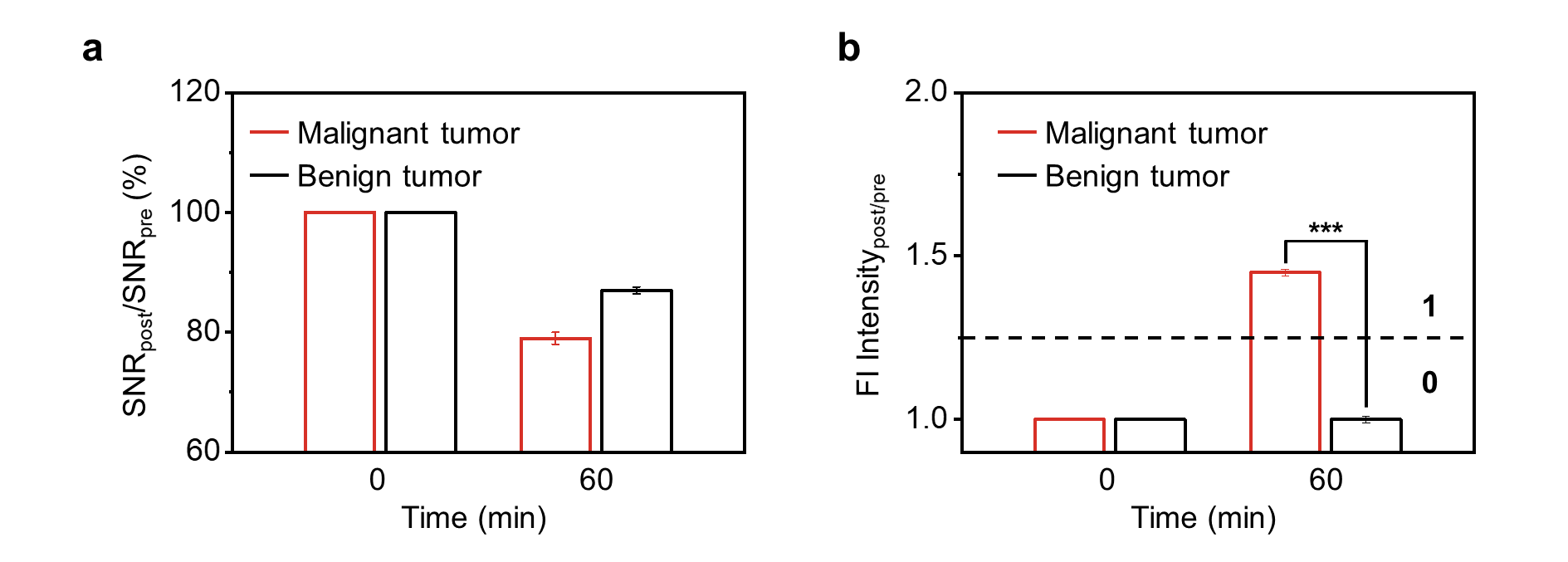


**Supplementary Fig. 8.** Quantification of dual-mode imaging signals before and after *i.v.* injection of KDMNs. Quantification of (a) *T*_2_ signal-to-noise ratios (SNRs) and (b) fluorescence intensities of tumors before and after *i.v.* administration of KDMNs. Data are presented as mean ± s.e.m. (n = 3). Data were compared using unpaired two-tailed Student’s t-tests. ^***^*P* = 0.0000257.

**
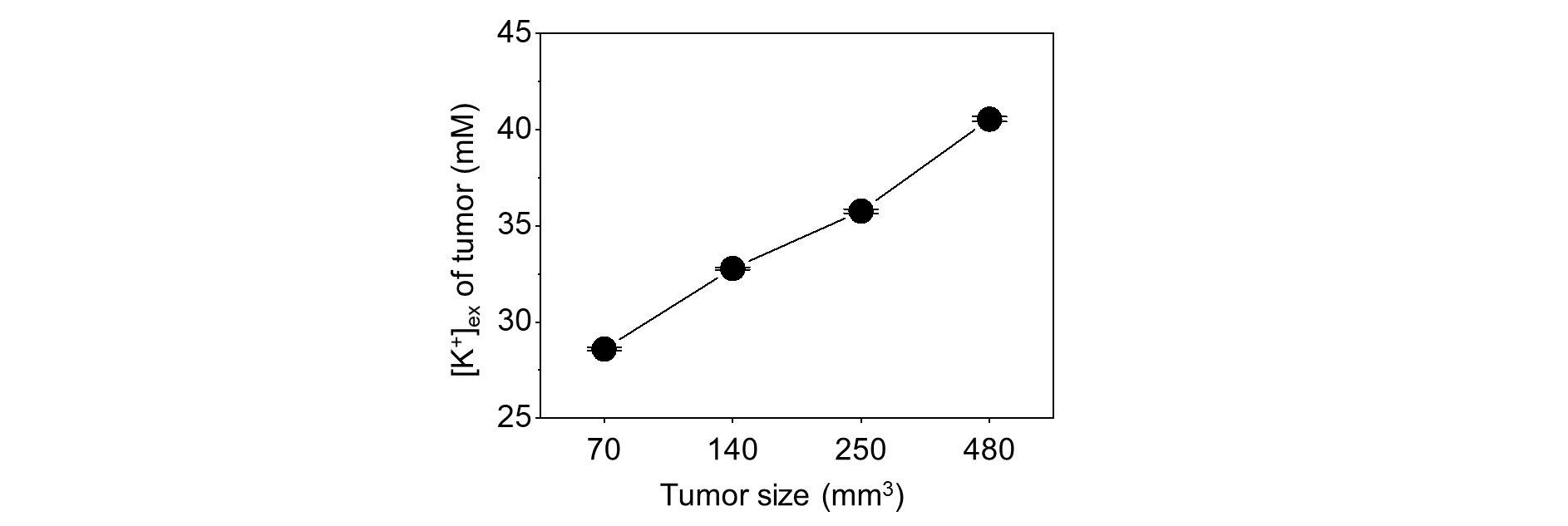
Supplementary Fig. 9.** [K^+^] in TIFs of malignant 4T1 tumors of different sizes. Data are presented as mean ± s.e.m. (n = 3).


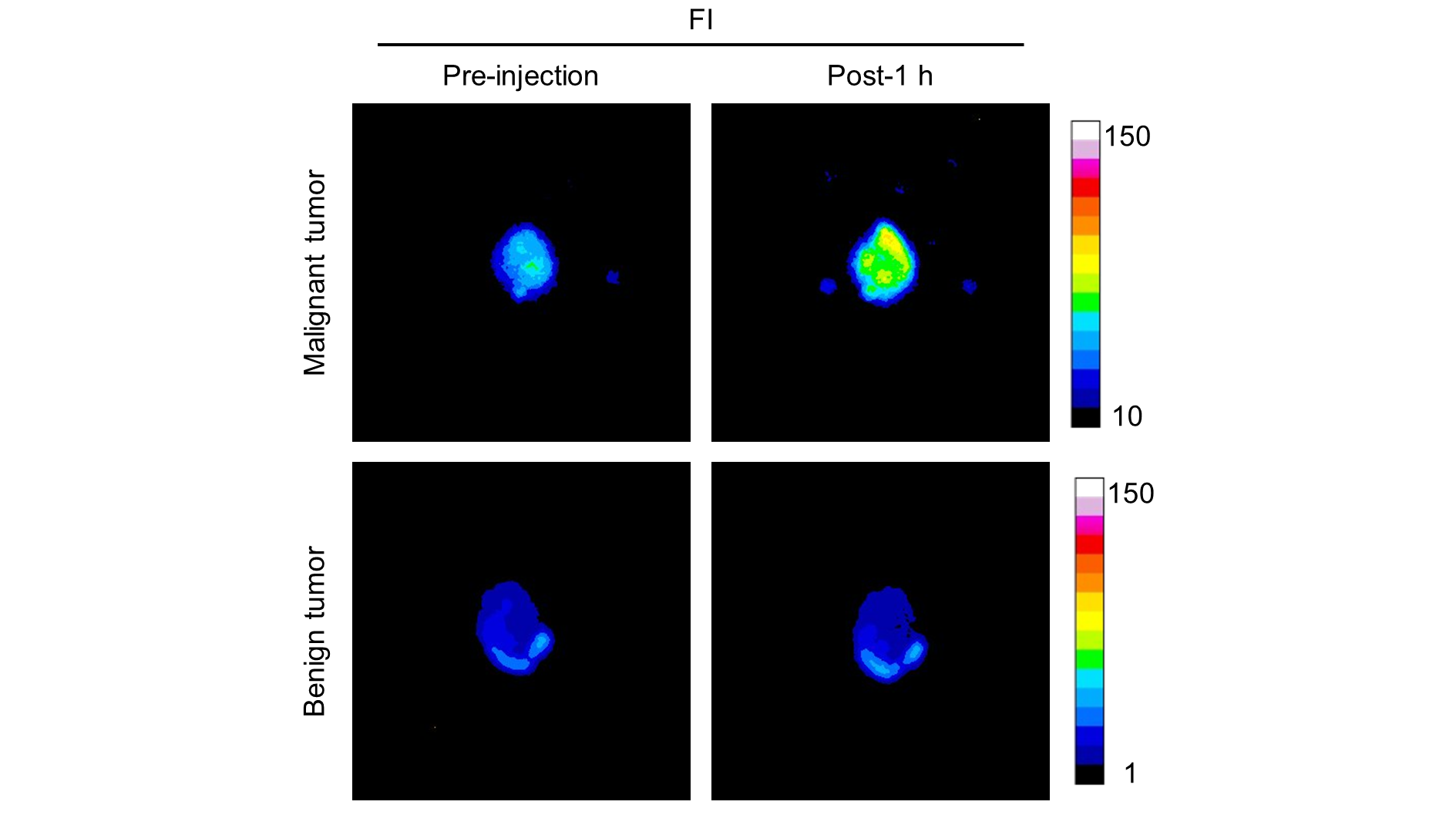


**Supplementary Fig. 10.** KDMN-based FI for malignancy identification in living mice. FI images of mice bearing malignant or benign xenografts before and at 1 h after *i.v.* injection of KDMNs.


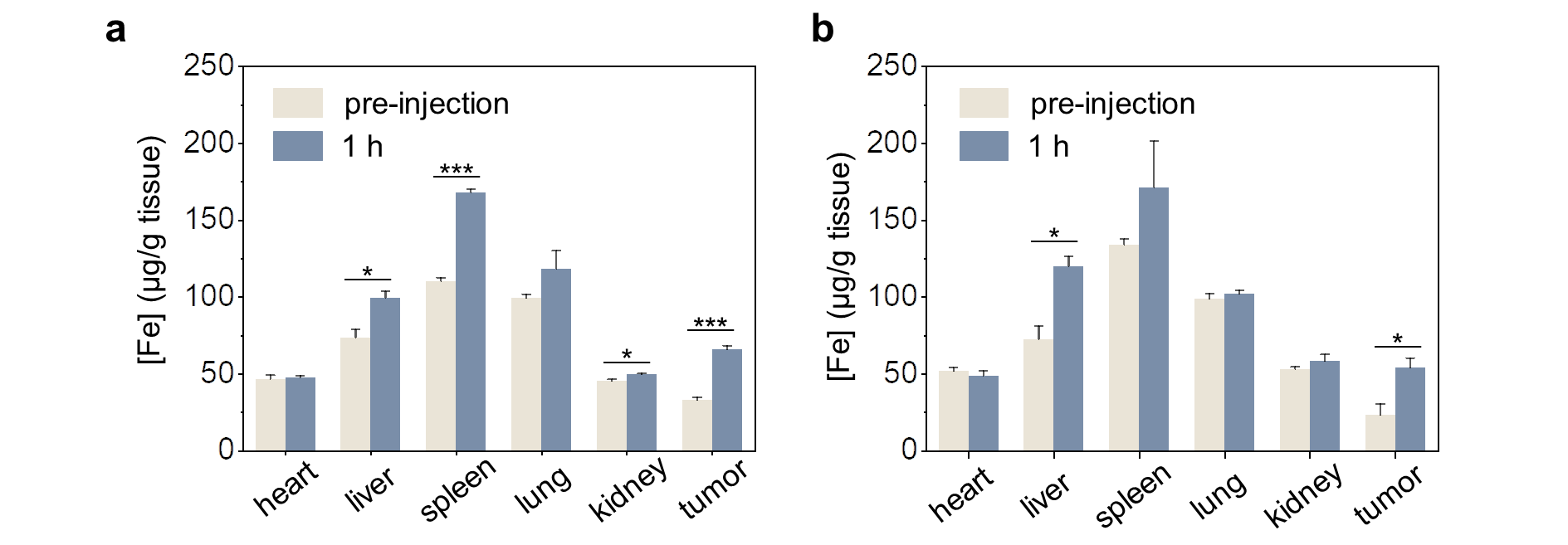


**Supplementary Fig. 11.** Tissue distributions of Fe before and after *i.v.* injection of KDMNs into mice bearing (a) malignant 4T1 or (b) benign human uterine leiomyoma xenografts. Data are presented as mean ± s.e.m. (n = 3). Data were compared using unpaired two-tailed Student’s t-tests, with *** indicating *p* < 0.001 and * indicating *p* < 0.05.


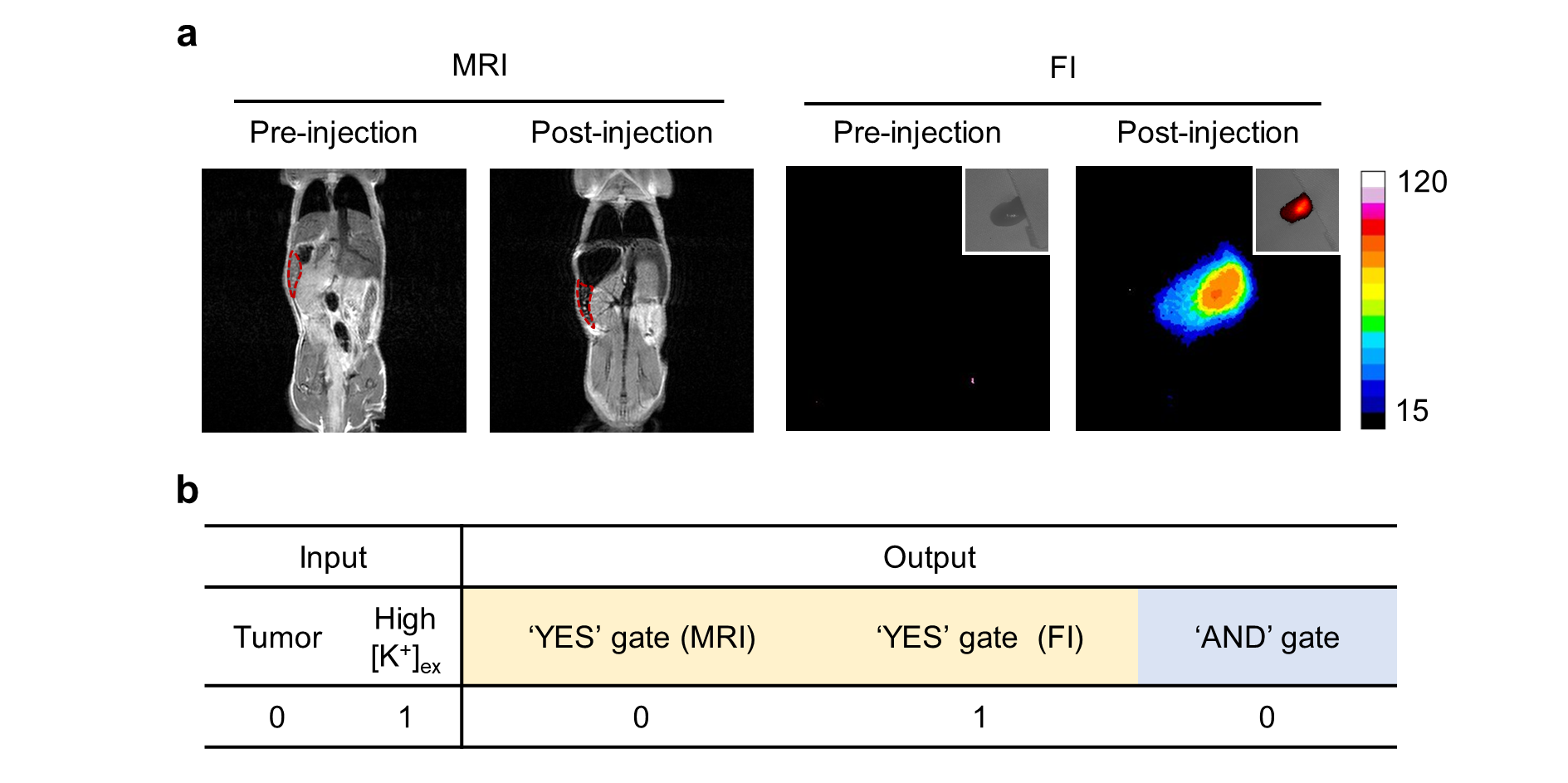


**Supplementary Fig. 12.** KDMN-based ‘AND’ logic dual-mode imaging for mice with high [K^+^]_ex_ in the spleen. The mice with high [K^+^]_ex_ in the spleen were constructed via intrasplenic injection of potassium chloride solution (50 mM, 50 *μ*L) after ligating the splenic vein and splenic artery. (a) MRI and FI images of mice with high [K^+^]_ex_ in spleen before and after intrasplenic injection of KDMNs. (b) The truth table of cascaded ‘AND’ logic gate for processing KDMN-based dual-mode imaging of mice with high [K^+^]_ex_ in the spleen.


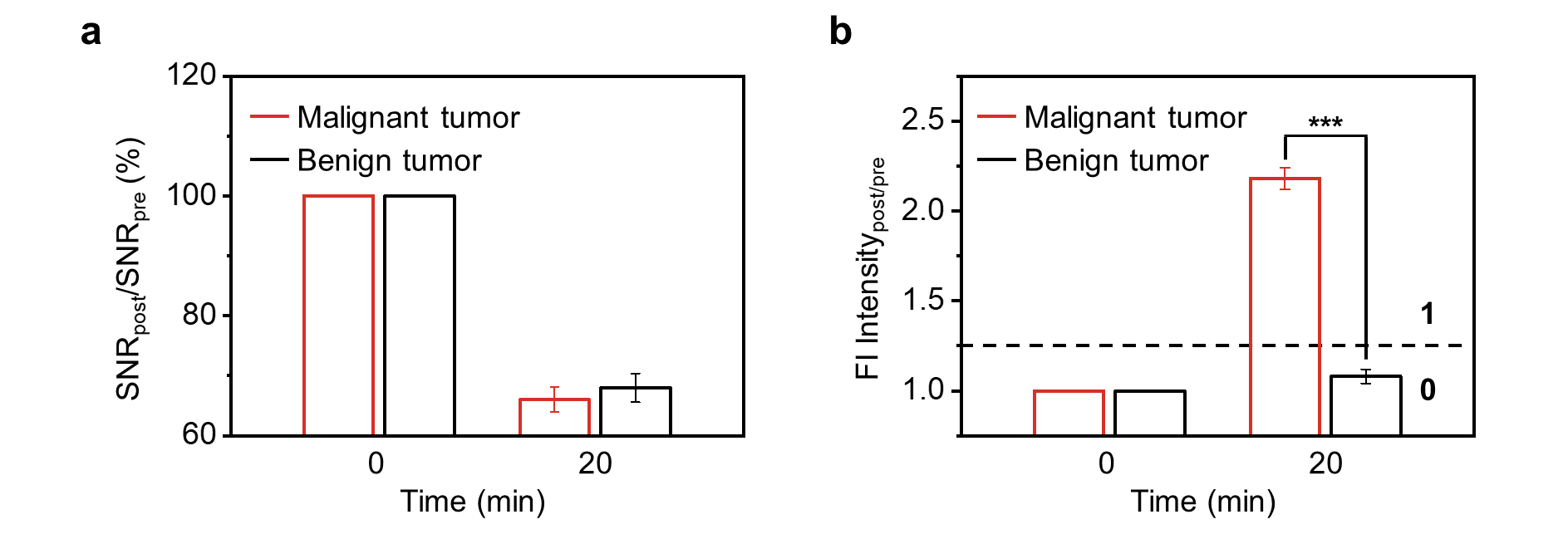


**Supplementary Fig. 13.** Quantification of dual-mode imaging signals before and after intratumoral injection of KDMNs. Quantification of (a) *T*_2_ SNRs and (b) fluorescence intensities of tumors before and after intratumoral administration of KDMNs. Data are presented as mean ± s.e.m. (n = 3). Data were compared using unpaired two-tailed Student’s t-tests. ^***^*P* = 0.0000895.


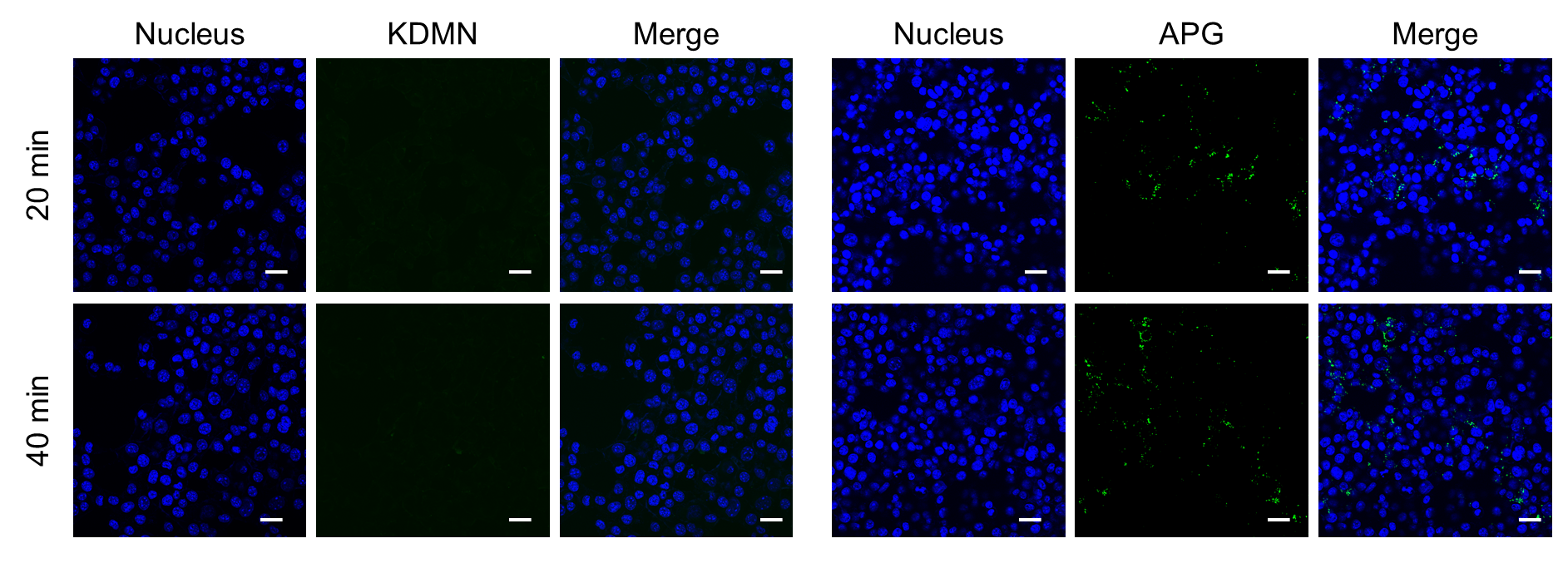


**Supplementary Fig. 14.** Cellular uptake of KDMNs and free APGs by 4T1 cells. Confocal laser scanning microscopic images of 4T1 cells incubated with KDMNs or free APGs for 20 and 40 min (scale bar = 30 *μ*m).
